# Comprehensive analysis of immunoglobulin expression in the mouse brain from embryonic to adult stages

**DOI:** 10.1186/s12974-025-03457-9

**Published:** 2025-06-09

**Authors:** Keiko Morimoto, Hitomi Sano, Michiko Takahashi, Rikuo Takahashi, Kazunori Nakajima

**Affiliations:** Department of Anatomy, Keio University School of Medicine, 35 Shinanomachi, Shinjuku-ku, Tokyo, 160-8582 Japan

**Keywords:** scRNA-seq, Immunoglobulin, *Ighm*, Constant region, Variable region, Neuron, Microglia, LPS

## Abstract

Immunoglobulin (Ig) is a critical molecule in the body’s defense against foreign substances such as bacteria and viruses. While traditionally considered B cell specific, studies have reported that Ig is also expressed by non-B cells. However, it is not known to what extent Ig is expressed in the brain and which type of variable regions are expressed. In this study, we elucidated the expression profile of Igs from embryonic to adult stages using single-cell RNA sequencing data and Ig repertoire analysis. Our results revealed that microglia express *Ighm* transcripts from embryonic to adult stages. These transcripts contain the upstream region of the *Ighj* region. In addition, *Ighm* is expressed in the layer 6 corticothalamic neurons, some of which co-express *Ighg2c* in the adult brain. In particular, we were able to generate a comprehensive profile of Ig variable region expression from embryonic to adult stages. Furthermore, the response of *Ighm* expression in microglia to lipopolysaccharide is markedly different from that in B cells, suggesting a novel, brain-specific role for the Ig gene, distinct from its classical function in the immune system.

**Supplementary Information:**

The online version contains supplementary material available at 10.1186/s12974-025-03457-9.

## Introduction

Antibodies are generally known as molecules that protect against infection by bacteria and viruses [1]. An antibody consists of two heavy chains and two light chains, each of which has a variable region (VDJ region for heavy chains and VJ region for light chains) and a constant region. Depending on the type of constant region, the following classes of immunoglobulins (Ig) are known in mice: IgM, IgD, IgG1, IgG2a (as found in BALB/c mice), IgG2b, IgG2c (as found in C57BL/6 and NOD mice), IgG3, IgE, and IgA.

The secreted form of Ig acts as an antibody and supports the immune response through functions such as antigen neutralization, opsonization, and complement activation. Antigen-antibody complexes are also taken up by phagocytic cells such as macrophages via specific receptors. For example, pIgR (polymeric immunoglobulin receptor), FcµR (Fc mu receptor), and Fcα/µR (Fc alpha and mu receptor) are known as receptors for IgM.

On the other hand, there are also membrane-bound Igs, which, together with the accessory molecules Igα (encoded by *Cd79a*) and Igβ (encoded by *Cd79b*), form the B cell receptors (BCRs). The signal from the membrane-bound Ig leads to B cell proliferation, differentiation, and antibody production, depending on co-stimulation and signal strength.

Previous studies have shown that immune molecules associated with acquired immunity, mediated primarily by B cells and T cells, are also expressed in the central nervous system (CNS), including Fcα/µR and major histocompatibility complex (MHC) class I molecules [2–7]. In addition, neurons and glial cells have recently been reported to express Ig themselves [8–12] in adulthood. As for the developing brain, the immature blood-brain barrier allows the entry of IgG of maternal origin [13–18]. These data suggest that Igs are present in the CNS from developmental stages into adulthood. However, these findings are still fragmentary and do not yet provide a complete picture of Ig dynamics in the brain.

Single-cell RNA sequencing (scRNA-seq) has greatly advanced our understanding of cellular heterogeneity and function by revealing gene expression profiles of individual cells. The gene expression data from the adult mouse cortex and hippocampus comprise approximately 1.1 million cells from male and female mice at approximately 8 weeks of age. By comparing these data with developmental RNA-seq data, we aim to explore changes in gene expression and cell types during brain development. The combination of scRNA-seq with bioinformatics tools such as Seurat [19] and multiple databases allows a detailed examination of developmental stages and cellular subclasses in the brain, providing a more comprehensive understanding of complex biological systems.

Therefore, we sought to fully elucidate the Ig gene expression by using scRNA-seq data and complementary molecular biology experiments.We identified the gene expression for the IgM heavy chain constant region (*Ighm*) with *Ighj* region in microglia, particularly during development, and in specific neuronal populations in the adult brain. During development, the IgG protein was of maternal origin only, but in the adult, some neurons expressed the RNAs for the constant regions of IgG and for multiple variable regions of the Ig heavy and light chains. The expression of Ig receptors, and their accessory molecules were also examined. We also investigated the response of microglia to lipopolysaccharide (LPS) stimulation, which mimics the bacterial infection and upregulates Ig expression in B cells, to gain insight into the potential function of *Ighm* expression in the brain.

## Materials and methods

### Experimental animals

Pregnant ICR and C57BL/6J mice were obtained from Japan SLC (Hamamatsu, Japan). The day of vaginal plug detection was considered as embryonic day (E) 0. Animal care and experiments were performed under the control of the Keio University Institutional Animal Care and Use Committee in accordance with the Institutional Guidelines on Animal Experimentation at Keio University, the Japanese Government Law on the Protection and Control of Animals, and the Japanese Government Notification of Feeding and Safekeeping of Animals.

### In situ hybridization and immunohistochemistry (IHC)

In situ hybridization was performed using in situ HCR v3.0 [20]. Mice were perfused with ice-cold 4% paraformaldehyde (PFA), and brains were dissected out and postfixed at 4 °C overnight. Brains were embedded in 3% low melting point agarose gels and 100 μm slices were prepared using a vibratome (VT1200 S, Leica Microsystems, Wetzlar, Germany). Staining steps were performed in a 48-well plate. Briefly, sections were incubated in a hybridization solution (Molecular Instruments, Los Angeles, CA) at 37 °C with shaking (a rotary shaker, 200 rpm). The probe set for mouse *Ighm* (20 probes were designed in the common sequences of the membrane-bound and secreted forms of the IgM constant region) was purchased from Molecular Instruments (Los Angeles, CA). Brain slices were incubated with 4 nM probes overnight at 37 °C with agitation. After washing with pre-warmed wash solution (Molecular Instruments, Los Angeles, CA) three times for 15 min at 37 °C with agitation, and with 5x SSC (Saline-Sodium Citrate buffer, 0.75 M NaCl, 0.075 M Sodium citrate pH 7.0) with 0.1% Tween20 (5x SSCT) three times for 5 min at room temperature (RT) with agitation, sections were incubated with fluorescence-labeled hairpins (B2-AlexaFluor488, B2-AlexaFluor647) reconstituted with an amplification solution (Molecular Instruments, Los Angeles, CA) overnight at RT with agitation. After washing three times with 5x SSCT for 5 min at RT with agitation, IHC was performed sequentially after fixation with 4% PFA for 10 min at RT. Samples were blocked with 10% normal goat or donkey serum in phosphate buffered saline (PBS) containing 0.05% Triton X-100 (PBST) for 1 h at RT and incubated with primary antibodies at 4  °C overnight. After three washes with PBST, samples were incubated with species-specific secondary antibodies and 4,6-diamidino-2-phenylindole, dihydrochloride (DAPI; Invitrogen, Waltham, MA, USA) (1/2000) for 2 h at RT. Brain slices were mounted on MAS-coated glass slides (MAS-02, Matsunami Glass, Osaka, Japan) using PermaFluor Aqueous Mounting Medium (TA-030-FM, Thermo Fisher Scientific, Waltham, MA, USA) and fluorescence images were captured with a confocal laser scanning microscope (TCS SP8; Leica Microsystems, Wetzlar, Germany).

The antibodies used in this study are listed below: anti-P2RY12 antibody (1:1000, AS-55043 A, Anaspec, Fremont, CA, USA), anti-FOXP2 antibody (1:1000, sc-21069, Santa Cruz, Biotechnology, Dallas, TX, USA), goat anti-rabbit IgG (H + L) cross-absorbed secondary antibody, Alexa Fluor™ 488 (1:1000, A11008, Invitrogen, Waltham, MA, USA), and donkey anti-goat IgG (H + L) cross-adsorbed secondary antibody, Alexa Fluor™ 555 (1:1000, A21432, Invitrogen, Waltham, MA, USA).

### RNA extraction and cDNA preparation

For reverse transcription polymerase chain reaction (RT-PCR) and quantitative real-time PCR analysis, total RNA was extracted from cells using TRIzol reagent (Thermo Fisher Scientific, Waltham, MA, USA) or TRIreagent LS (Cosmo Bio Co., Ltd., Tokyo, Japan) or Sepasol RNA I Super G (Nacalai Tesque, Inc., Kyoto, Japan) according to the manufacturer’s instructions. cDNA was synthesized using Superscript III First-strand Synthesis System for RT-PCR (Thermo Fisher Scientific, Waltham, MA, USA) or ReverTraAceqPCR RT Master Mix with gDNA remover (TOYOBO Co., Ltd., Osaka, Japan).

### Reverse transcription polymerase chain reaction (RT-PCR)

RT-PCR was performed using Ex Taq DNA Polymerase (Takara Bio Inc., Shiga, Japan) according to the manufacturer’s instructions. The following pair of primers was used:

Membrane-bound form: sense 5’- AAGTACCTAGCCACCTCGCA − 3’ and antisense 5’- TGGGCCAGACATTGCTTCAG − 3’, secreted form: sense 5’- AAGTACCTAGCCACCTCGCA − 3’ and antisense 5’- AATAGCAGGTGCCGCCTGTG − 3’, transcript with intergenic region: sense 5’- TGCAAGCATCAGAAGGGTGG − 3’ and antisense 5’- GCTAGGTACTTGCCCCCTGT − 3’, *Cd19*: sense 5’-GCCACAGCTTTAGATGAAGGCAC − 3’ and antisense 5’- CATCCACCAGTTCTCAACAGCC − 3’. *Cd19* primer sequences were the same as the Origine primer sets (MP201884). PCR amplification was performed using a Veriti 96-Well Thermal Cycler (Applied Biosystems, Thermo Fisher Scientific, Waltham, MA, USA) under the following conditions: initial denaturation at 95 °C for 1 min, followed by 35 cycles of denaturation at 95 °C for 30 s, annealing at 55 °C for 30 s, and extension at 72 °C for 90 s (30 s for *Cd19*), with a final extension at 72 °C for 7 min. The PCR products were analyzed by agarose gel electrophoresis (1.5% agarose, 135 V, 30 min) and visualized with a FAS-V imaging system (Nippon Genetics Co., Ltd., Tokyo, Japan).

### Quantitative real-time PCR analysis

To quantify *Ighm* RNAs, quantitative real-time PCR was performed on a QuantStudio 5 Real-Time PCR System (Applied Biosystems, Thermo Fisher Scientific, Waltham, MA, USA). Cycling conditions consisted of an initial denaturation at 95°C for 30 s, followed by 40 cycles of denaturation at 95°C for 15 s and annealing/extension at 60°C for 30 s. The following primer pairs were used: *Actb*, sense 5’- AGTGTGACGTTGACATCCGTA − 3’ and antisense 5’- GCCAGAGCAGTAATCTCCTTCT − 3’; *Ighm*, sense 5’- GTGGATCACAGGGGTCTCA − 3’ and antisense 5’- GTCAGGTTAGCGGACTTGCT − 3’.

### Flow cytometry

Freshly prepared brain samples were minced into 1–2 mm pieces and incubated for 19 min for P0 brain and 30 min for adult brain at 37 °C in the presence of 10 units/ml papain (cat#26035-44, Nacalai Tesque, Inc., Kyoto, Japan) and 0.2 mg/ml DNase (DN25-100 mg, Sigma-Aldrich, St. Louis, MO, USA) in Dulbecco’s phosphate buffered saline (D-PBS(-)) containing 0.5 mg/ml bovine serum albumin, 0.5 mg/ml Cystein and 5 mg/ml D-glucose. After neutralization of papain with fetal bovine serum, the sample was pipetted and filtered through a 70-µm pore size nylon mesh. The cell suspension in 30% Percoll was centrifuged at 500×g for 25 min to remove myelin components. The cell pellet was used for fluorescence-activated cell sorting (FACS). The cells were stained with the following antibodies: Alexa Fluor^®^ 488 rat anti-mouse CD11b (clone M1/70, cat#557672, BD Pharmingen, San Jose, CA, USA,1:200), APC anti-mouse CD45 antibody (30-F11, cat# 103112, Biolegend, San Diego, CA, USA, 1:200), APC anti-human/mouse CD45R (B220) (RA3-6B2, cat#20–0452, TONBO Biosciences, San Diego, CA, USA). Cells were sorted by MoFlo XDP (Beckman Coulter, Brea, CA, USA) and data were analyzed using FlowJo software version 10.10.0 (BD Biosciences, Franklin Lakes, NJ, USA).

### Lipopolysaccharide (LPS) injection model

Male 9 W ICR mice were injected intraperitoneally with 250 µg/kg LPS from Escherichia coli 055:B5 (L2880, Sigma-Aldrich, St. Louis, MO, USA) [21], and the whole brains without cerebellum were harvested 20 h later.

### Microglia culture

CD11b + CD45 + microglia obtained by FACS from P0 ICR mice were cultured in Dulbecco’s Modified Eagle Medium, high glucose, pyruvate (cat#11995-065, Gibco, Waltham, MA, USA) containing 10% fetal bovine serum at a density of 5 × 10^4^ cells per 100 µl medium at 37 °C overnight. The RNAs were prepared after 5 h of stimulation with 1 µg/ml LPS from Escherichia coli 055:B5 (L2880, Sigma-Aldrich, St. Louis, MO, USA).

### Ig repertoire analysis

Cortical cells were obtained from three P0 C57BL/6J mice. Briefly, the cortex was dissected after transcardiac perfusion with D-PBS (-) to remove peripheral blood cells. A single-cell suspension was prepared using papain (Nacalai Tesque Inc., Kyoto, Japan), following a protocol similar to that used for flow cytometry sample preparation, and approximately 1.7 × 10^4^ cells were harvested. The scRNA-seq and Ig libraries were generated using the Chromium Next GEM Single Cell V(D)J Reagent Kit v1.1 (10x Genomics, Pleasanton, CA, USA) according to the manufacturer’s protocol. Sequencing was performed on a NovaSeq 6000 platform (Illumina, San Diego, CA, USA). Ig repertoires were analyzed using the Cell Ranger VDJ pipeline (version 3.1.0) in de novo mode. Raw FASTQ files were processed to identify and assemble contigs without the use of a predefined reference; contig sequences were extracted and filtered based on the quality criteria.

### Single cell data analysis using public-domain databases

To analyze embryonic stages, we used data published by Manno et al., (2021) [22] (PRJNA637987). They presented a comprehensive single cell transcriptomic atlas of the developing mouse brain from gastrulation to birth. We imported the data as a Seurat object using the Seurat library version 4. Data from E16.0 to 18.0 were extracted, and cells belonging to four specific classes (bad cells, ectoderm, gastrulation, and mesenchyme) were excluded from the dataset. Specifically, we applied the NormalizeData function with the method = “LogNormalize”, which normalizes the data by dividing the raw gene expression counts by the total counts per cell, multiplying by a scaling factor of 10,000, and then log-transforming the result. No additional normalization steps were applied. Batch effects were not considered in this analysis, and no batch correction methods were implemented.

In order to study the *Ig* in the adult stage, we used the data published by Yao et al., (2021) [23] (PRJNA772116). This “Mouse Whole Cortex and Hippocampus 10x” data set (https://portal.brain-map.org/atlases-and-data/rnaseq/mouse-whole-cortex-and-hippocampus-10x*)* contains single-cell transcriptomes from multiple cortical areas and the hippocampal formation, totaling ~ 1.1 million cells; samples were collected from brain region dissections of ~ 8-week-old male and female mice of many transgenic mouse lines or mice labeled with different viral vectors. Single cell data were processed and visualized using custom Python code and the Seurat R package (version 4) [24].

### Statistical analysis

Quantitative data are presented as mean ± standard deviation (SD). Individual values are shown as circles in bar graphs. Statistical analysis was performed using Welch’s *t*-test. Differences with *P* values < 0.05 were considered significant. ***, *P* < 0.001.

## Results

### Detection of *Ighm* RNA expression in the embryonic brain by scRNA-seq data analysis

**Fig. 1:**
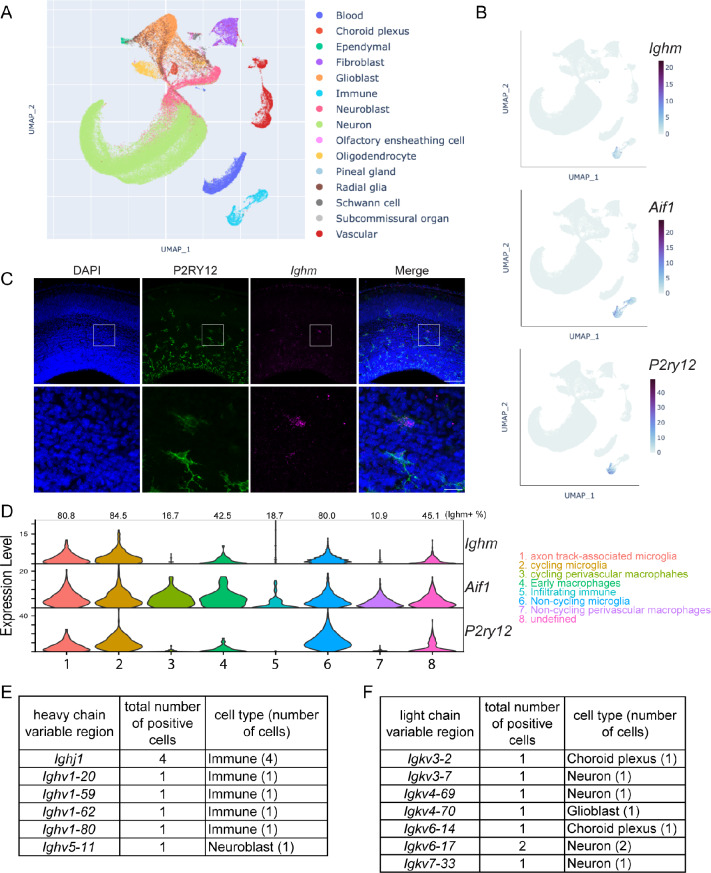
Identification of *Ighm* expression in microglia at embryonic stages. **A**. UMAP visualization using scRNA-seq data from E16.0-E18.0 mouse brain based on PRJNA637987. 15 clusters were visualized. **B**. The expression of *Ighm*, *Aif1* (microglia and macrophage - common marker) and *P2ry12* (microglia specific marker) is enriched in Immune cluster. Color intensity indicates expression level of normalized UMI (Unique Molecular Identifier) counts. **C**. In situ hybridization combined with IHC of E16 cerebral cortex. P2RY12 positive microglia express *Ighm*. Scale bar = 100 μm, 20 μm. *n* = 2 brains. **D**. Violin plot of *Ighm*, *Aif1* and *P2ry12* of immune cluster. The cluster was subdivided into 8 classes. Values on the top of the plot represent the percentage of cells with *Ighm*-positive. **E**, **F**. Cell types expressing the variable regions of the Ig heavy (**E**) and light (**F**) chains in the embryonic database

To comprehensively investigate the expression of Ig-related genes, we decided to analyze a published database PRJNA637987 and examined the expression in the embryonic mouse brain (E16.0-E18.0). A uniform manifold approximation and projection (UMAP) plot revealed 15 cell classes (Fig. 1A), for which the expression of constant and variable regions of Igs were examined (Table S1). Among them, *Ighm* was abundantly detected in the immune cell class (Fig. 1A and B). To examine the distribution of microglia and perivascular macrophages, the predominant immune cells in the brain, we visualized the expression of *Ighm* and *Aif1* (encoding IBA1), which are expressed in both cell types, as well as *P2ry12*, a microglia-specific marker, using a UMAP plot (Fig. 1B). In situ hybridization also confirmed that purinergic receptor P2Y, G-protein coupled 12 (P2RY12)-positive microglia were *Ighm*-positive in the cerebral cortex of E16 mice (Fig. 1C). The immune cell class was further subdivided into 8 subclasses based on the classification reported in the original paper, and more than 80% of microglia (axon track-associated microglia, cycling microglia, and non-cycling microglia) and less than half of macrophages (cycling perivascular macrophages, early macrophages, and non-cycling perivascular macrophages) expressed *Ighm* (Fig. 1D). Although the majority of microglia expressed RNAs for the constant region of the IgM heavy chain, RNAs for the variable regions of the Ig heavy chain were detected in only a few immune cells; *Ighj1* (4 cells), *Ighv1-20* (1 cell), *Ighv1-59* (1 cell), *Ighv1-62* (1 cell), *Ighv1-80* (1 cell) (Fig. 1E, Table S1). Several variable regions of the Ig light chain, including *Igkv3-2* (1 cell), *Igkv3-7* (1 cell), *Igkv4-69* (1 cell), *Igkv4-70* (1 cell), *Igkv6-14* (1 cell), *Igkv6-17* (2 cells), and *Igkv7-33* (1 cell), were also detected in this database containing 97,075 cells (Fig. 1F, Table S1). One of the constant regions, *Igkc*, was detected in one cell (glioblast).

### Expression and characterization of Ig transcripts in the neonatal brain

**Fig. 2:**
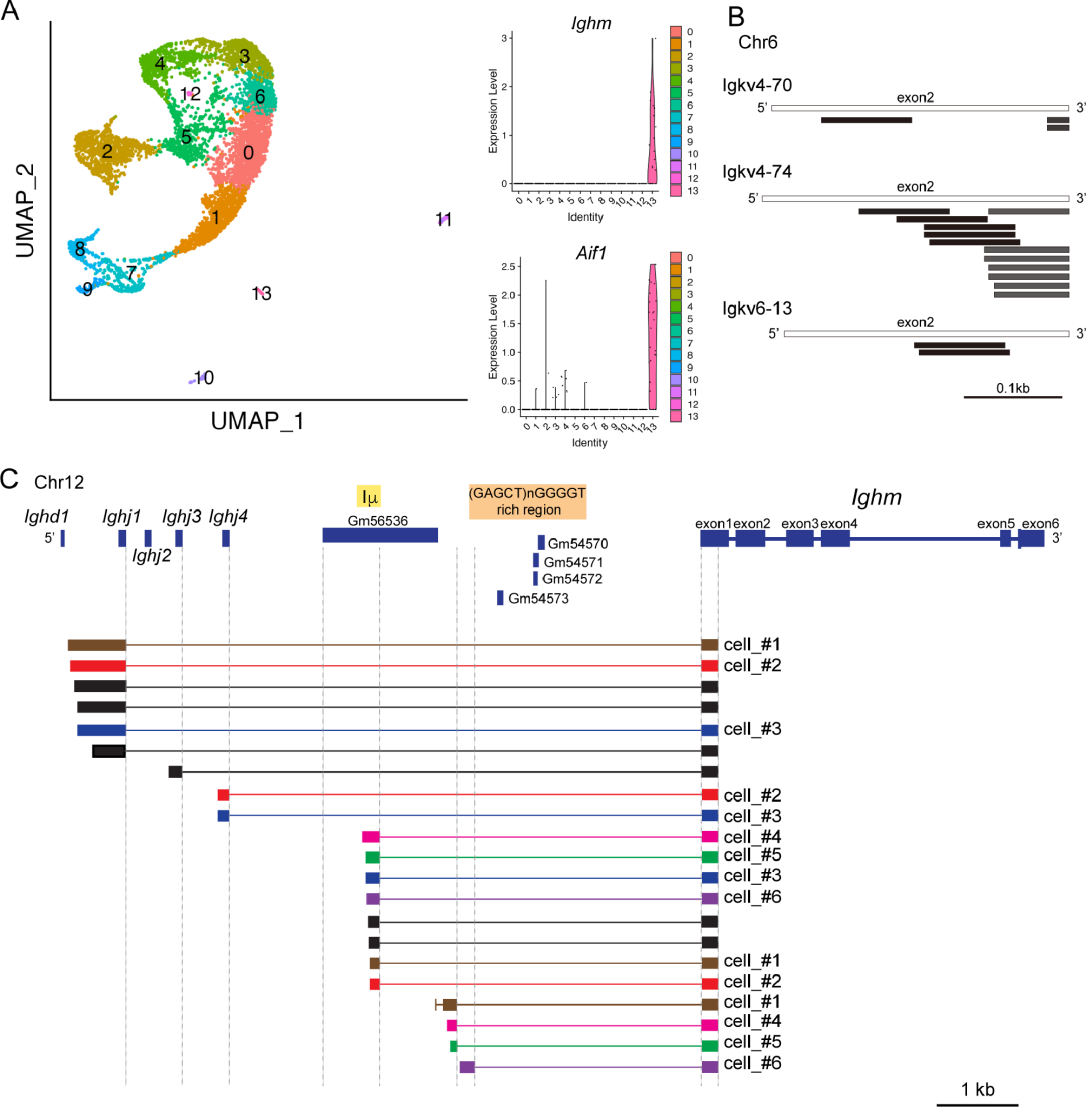
Immunoglobulin transcripts in the neonatal brain. **A**. UMAP visualization and violin plots using scRNA-seq data of P0 cerebral cortex. 14 clusters were identified and *Ighm* and *Aif1* were highly expressed in cluster 13. Normalized UMI (Unique Molecular Identifier) counts were used to represent the expression level. **B**. Variable Reads (dark color lines) were mapped to *Igkv4-70*, *Igkv4-74*, and *Igkv6-13* exons (light color lines); each *Igkv* region consists of 2 exons. **C**. Read sequences were assembled to contigs, which were mapped to genomic region of upper region of *Ighj1* to *Ighm*. Colors and cell numbers represent that the contigs were originated from the same cells

To gain a deeper understanding of the expression of Ig variable regions in these cells, we performed Cell Ranger V(D)J analysis using our own dataset of P0 mouse brain. Among the 14 cell clusters identified from 6,282 P0 cerebral cortical cells, cluster 13 was distinguished by its high expression of *Aif1* (Fig. 2A). Notably, cluster 13 exhibited high *Ighm* expression; out of 26 cells, 15 cells (58%) were *Ighm* positive. The expression levels of all other Ig constant and variable regions are shown in Table S2. We detected RNAs for three different variable region genes across three different cells within the dataset (Fig. 2B). *Igkv4-70* and *Igkv4-74* were identified in cluster 4 cells, and *Igkv6-13* was identified in a cluster 8 cell. The markers for each cluster obtained by the FindMarker function are *Sox5*, *Fezf2*, *Crym*, *Tle4*, *Igfbp3* for cluster 4, and *Aldoc*, *Tnc*, *Mfge8*, *Phgdh*, and *Mt1* for cluster 8. For more detailed cell type identification, analysis was performed using MapMyCells (RRID: SCR_024672) (https://portal.brain-map.org/atlases-and-data/bkp/mapmycells), which revealed that the *Igkv4-70* positive cell was OB-STR-CTX Inh IMN (olfactory bulb-striatum-cerebral cortex inhibitory immature neuron grouped with GABAergic neuron subclasses in OB [25]), the *Igkv4-74* positive cell was L6 CT CTX Glut (Layer 6 corticothalamic, cerebral cortex, glutamatergic neuron), and the *Igkv6-13* cell was Astro-TE NN (astrocytes located in telencephalic regions, non-neuronal).

To further analyze the variable regions with low expression levels, Ig repertoire analysis was performed, but no contig containing the entire V(D)J region was obtained. However, transcripts that linked the J region to the *Ighm*, the constant region of IgM heavy chain, were detected (Fig. 2C). Interestingly, the upstream intergenic region of the *Ighj1*, *Ighj3* and *Ighj4* was transcribed. In addition, *Ighj1* and *Ighj4* were simultaneously expressed in two cells, and a germline transcript, Iµ-Cµ, was also detected in these data. Transcripts starting from the region between Iµ and the (GAGCT)nGGGGT-rich switch region were also detected. Importantly, *Ighm*-positive cells were negative for *Cd19*, *Pax5*, and *Ebf1*, which are pan-B cell markers. *Prdm1* (encoding Blimp1, a plasma cell marker) was also negative. These results confirm that the *Ighm*-containing transcripts we detected were not derived from contaminated B cells.

**Fig. 3:**
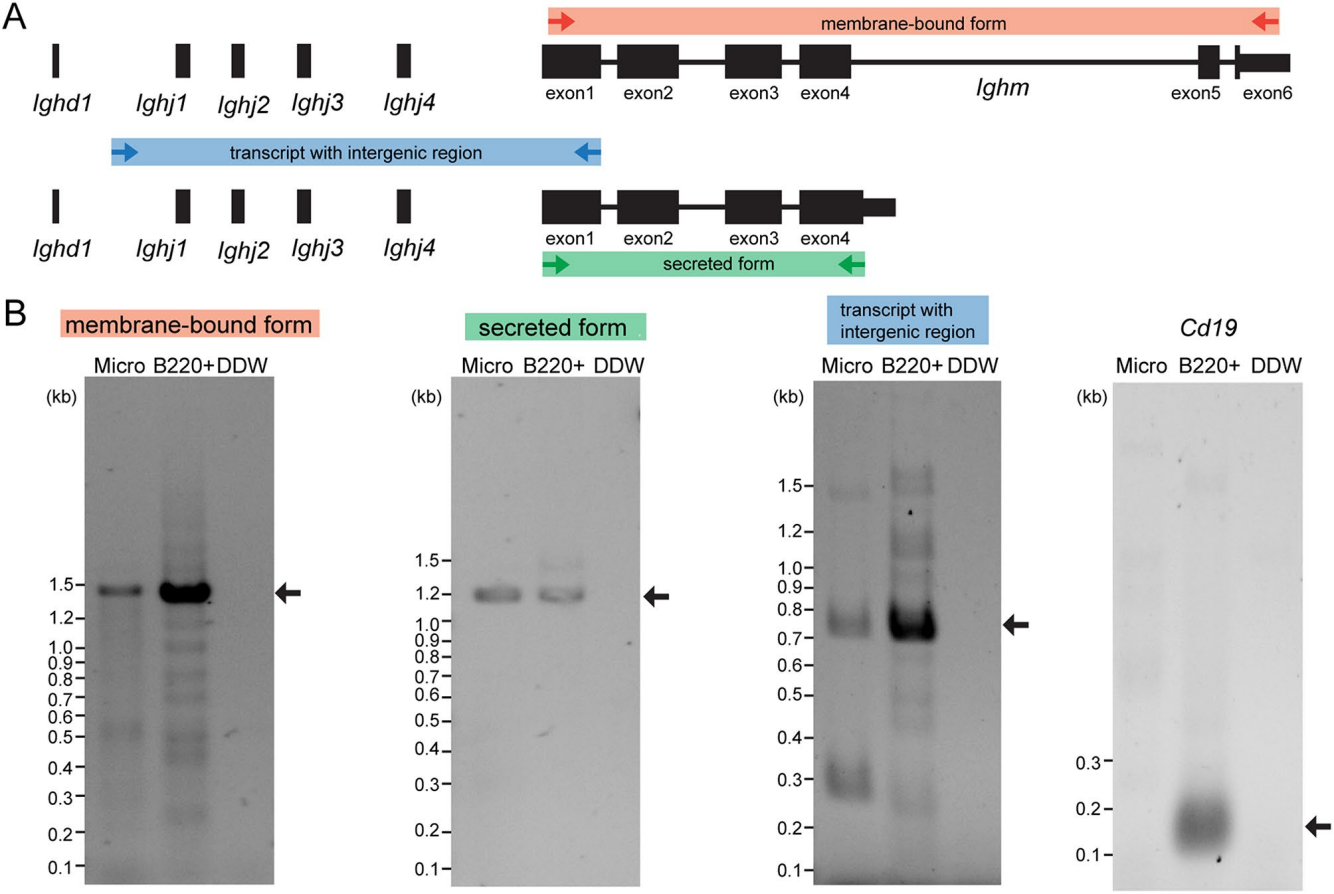
PCR of *Ighm* transcripts in microglia. **A**. Three pairs of primer sets are illustrated on the genomic regions of the *Ighm*. **B**. Membrane-bound form, secreted form, and transcript with intergenic region were detected by PCR on microglial samples (FACS-sorted CD11b + CD45 + cells) from P0 mice and FACS-sorted B220 (CD45R)+  cells (containing B cells, plasmacytoid dendritic cells, etc.) from 8-week-old mice . *n* = 3 for each sample. Sequences were confirmed by Sanger sequencing. Micro: microglia. PCR for *Cd19* was performed to exclude the possibility of B cell contamination in the microglial sample. Arrows indicate expected PCR products

Since it was unclear from the scRNA-seq data whether the membrane-bound form or secreted form is transcribed from the *Ighm* region, we performed RT-PCR. The membrane-bound form is encoded by exons 1–6 of *Ighm*, while the secreted form is encoded by its exons 1–4. As a result, both membrane-bound and secreted form transcripts were detected in CD11b + CD45 + microglia of P0 brains sorted by FACS (Fig. 3A, B). The presence of transcripts containing the upstream sequence of *Ighj1* and extending to the *Ighm* (transcript with intergenic region) was also confirmed by RT-PCR. *Cd19* RNA was not detected by RT-PCR in the microglial sample. These data confirmed that microglia express both membrane-bound and secreted forms of *Ighm* and that the 5’ upstream region (intergenic sequence) of *Ighj*1 gene is indeed transcribed.

### Ig expression patterns in the adult brain

**Fig. 4:**
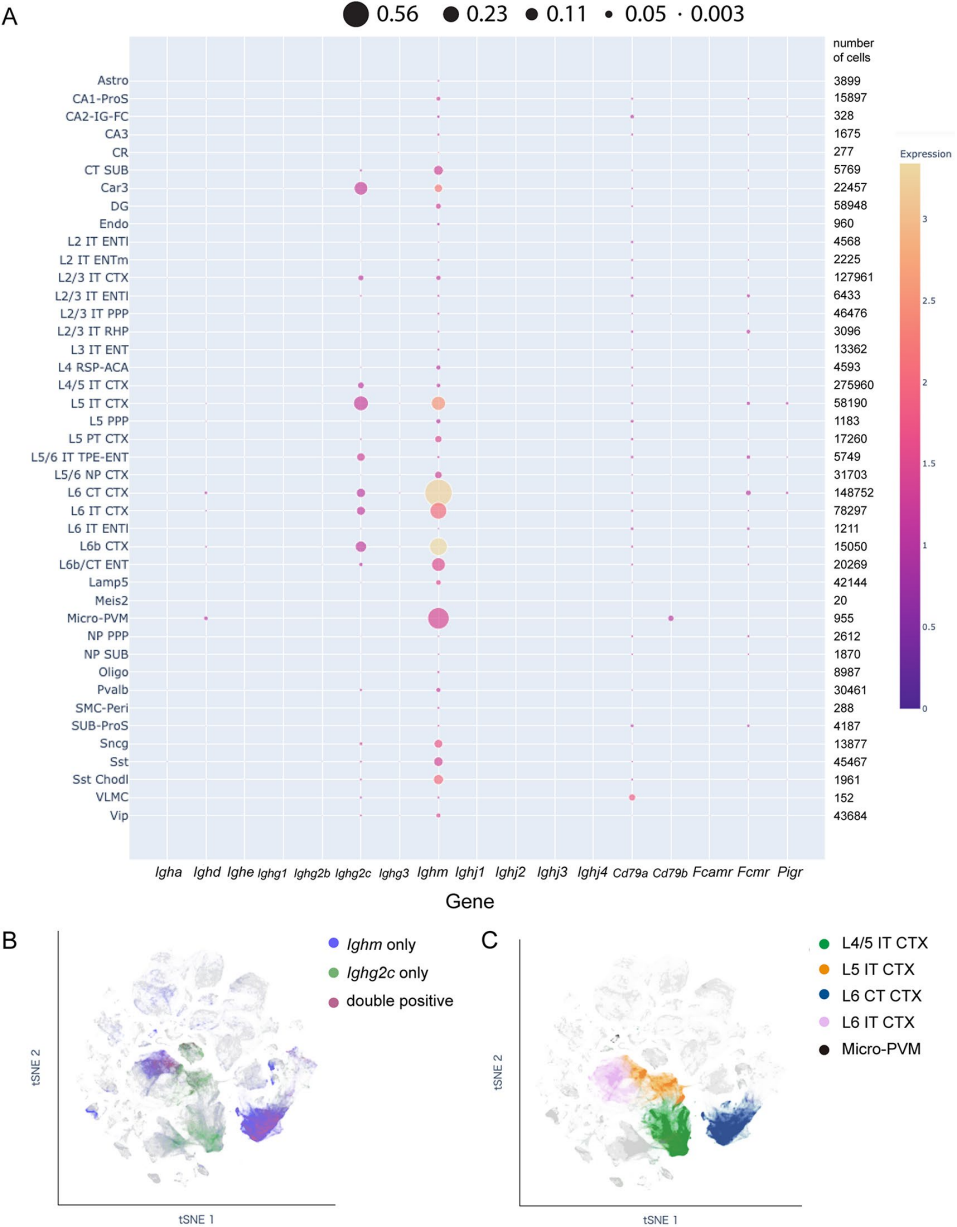
Analysis of Ig transcripts in adult scRNA-seq database. **A**. Dot plots of genes including *Ig* constant region and *Ighj* region, components of membrane-bound IgG receptors (*Cd79a*, *Cd79b*) and IgM receptors (*Pigr*, *Fcmr*, *Fcamr*) in 42 subclasses based on PRJNA772116. The size of the dot corresponds to the percentage of positive cells in the subclass. Larger dots indicate a higher fraction of positive cells, while smaller dots represent lower proportions. The color of the dot is determined by a color scale, where the hue indicates the average expression level (counts per million: CPM) of the target (from low to high expression). On the right side of the plot, the total number of cells in each subclass is displayed. **B**. t-SNE projection of *Ighm*-only, *Ighg2c*-only and double-positive cells. **C**. L4/5 IT CTX, L5 IT CTX, L6 CT CTX, L6 IT CTX and Micro-PVM cells are mapped on the t-SNE plot. ACA: Anterior cingulate area, Astro: astrocyte, Car3: carbonic anhydrase 3, Chodl: chondrolectin, CR: Cajal-Retzius, CT: corticothalamic-projecting glutamatergic cortical neuron, CTX: Cerebral cortex, DG: Dentate gyrus, Endo: endothelial cell, ENT: Entorhinal area, ENTl: Entorhinal area, lateral part, ENTm: Entorhinal area, medial part, FC: fasciola cinereal, IG: indusium griseum, IT: intratelencephalic-projecting glutamatergic cortical neuron, Lamp5: lysosomal-associated membrane protein family, member 5, L2: cortical layer 2, L3: cortical layer 3, L4: cortical layer 4, L5: cortical layer 5, L6: cortical layer 6, Meis2: Meis homeobox 2, Micro: microglia, NP: near-projecting glutamatergic cortical neuron, Olig: oligodendrocyte, Peri: pericyte, PPP: postsubiculum-presubiculum-parasubiculum, ProS: prosubiculum, PT: Pyramidal tract, Pvalb: parvalbumin, PVM: perivascular macrophage, RHP: Retrohippocampal region, RSP: Retrosplenial area, SMC: smooth muscle cell, Sncg: synuclein, gamma, Sst: somatostatin, SUB: Subiculum, TPE: temporal association-perirhinal-ectorhinal area, Vip: vasoactive intestinal polypeptide, VLMC: vascular leptomeningeal cell. See the original paper [23] for detailed annotation information

The expression of Ig regions in the adult brain was also investigated using the public scRNA-seq database of the entire adult mouse isocortex and hippocampus, focusing on glutamatergic and GABAergic neuron types (Fig. 4A, Table S3). Visualization of the percentage of positive cells per cell subclass revealed that not only *Ighm* but also *Ighg2c* was highly expressed in the adult brain. While positive spots were not visible for *Igha*, *Ighe*, *Ighg1*, and *Ighg2b* in Fig. 4A, they were also detected in a few cells (Table S3). Approximately 56% of layer 6 corticothalamic neurons in the cerebral cortex (L6 CT CTX), 35% of microglia/perivascular macrophages (Micro-PVM), and 21% of layer 6 intratelencephalic neurons in the cerebral cortex (L6 IT CTX) were positive for *Ighm* (Fig. 4A, Table S4). In contrast, few cells in the superficial layers of cerebral cortex expressed *Ighm*. In situ hybridization confirmed that *Ighm* is expressed in the deep layer of the cerebral cortex, where FOXP2-positive corticothalamic neurons express *Ighm* (Fig. S1A, S1B). On the other hand, *Ighm* expression in microglia could not be clearly detected by in situ hybridization (data not shown).

UMAP projection showed that *Ighm* and *Ighg2c* double positive cells were mainly observed in L6 CT CTX, L5 IT CTX and L6 IT CTX, and *Ighg2c* single positive cells were observed in L4/5 IT CTX (Fig. 4B and C, S2).

**Fig. 5:**
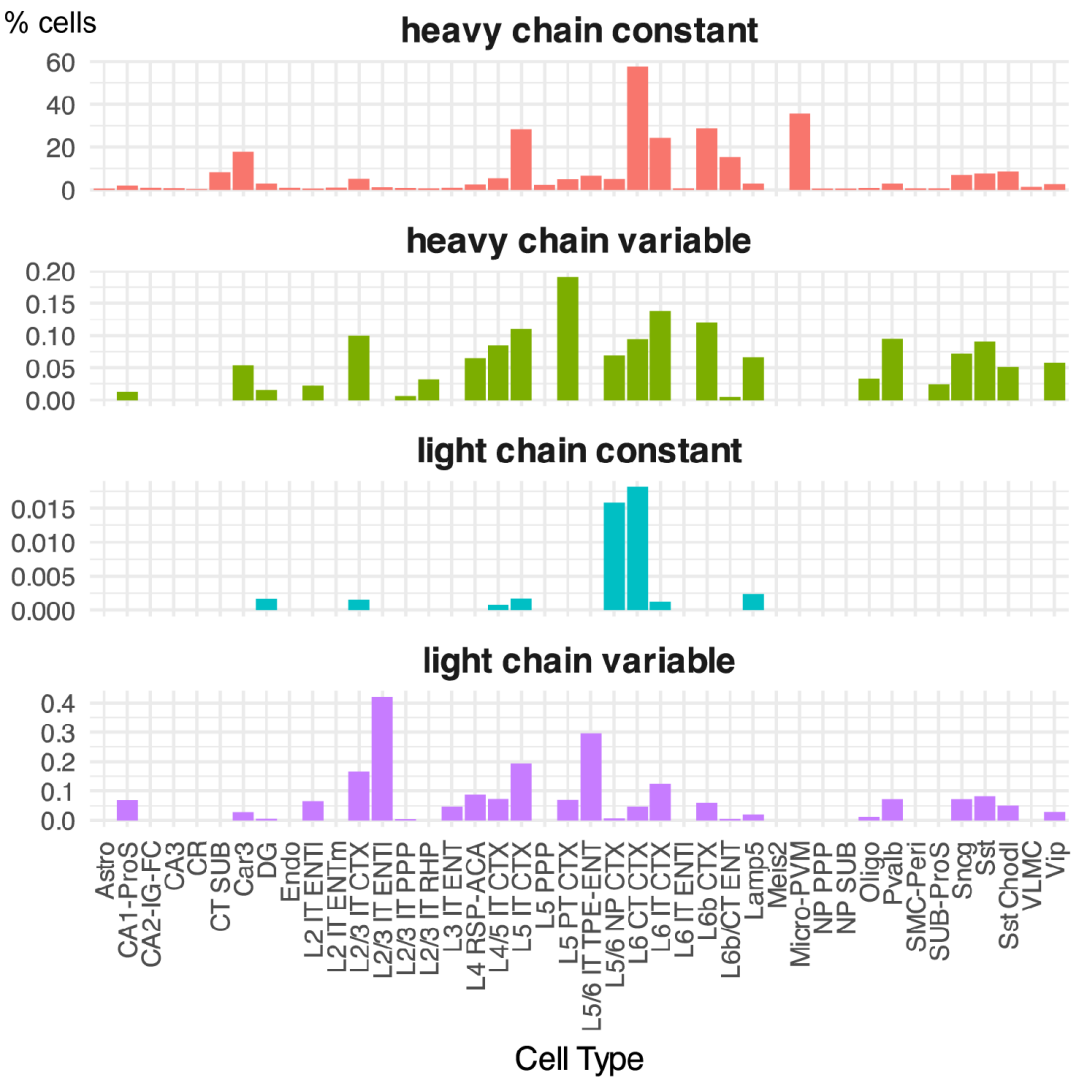
Proportion of cells that express variable/constant regions of the Ig light/heavy chains in the adult database. Bar graph showing the percentage of cells expressing any of the Ig heavy chain constant region, Ig heavy chain variable region, Ig light chain constant region, or Ig light chain variable region in each cell type calculated from the adult scRNA-seq data. For abbreviations, see the legend to Fig. 4

**Fig. 6:**
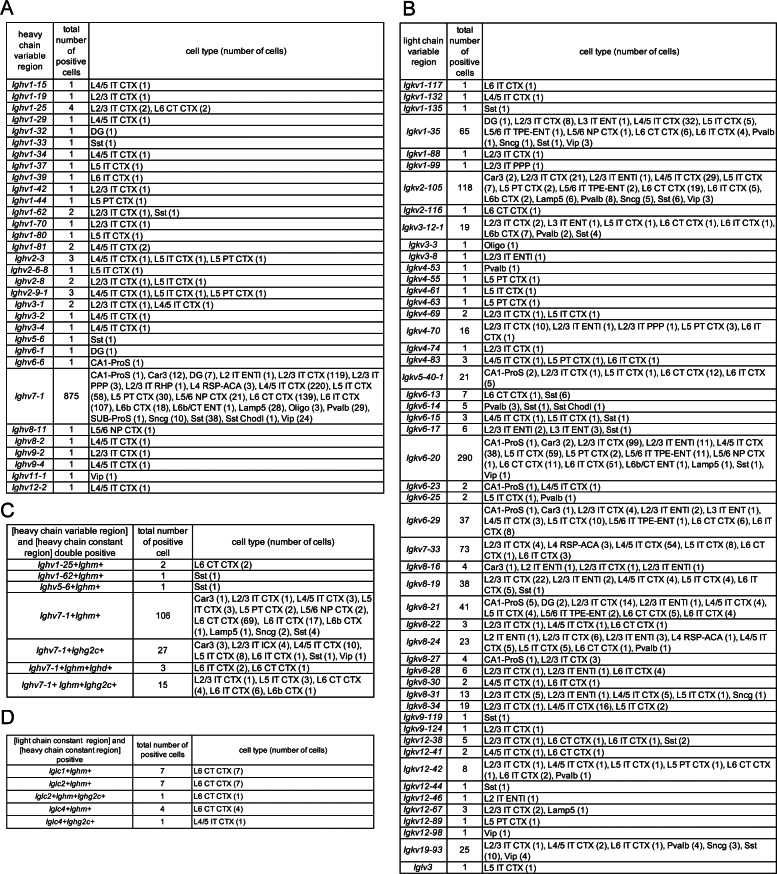
Expression of the Ig variable regions in the cerebral cortex and hippocampal formation of adult mice. **A**, **B**. Cell types expressing the variable regions of the Ig heavy (**A**) and light (**B**) chains in the adult database. **C**. Cells expressing both the variable and constant regions of Ig heavy chains. **D**. Cells expressing both the Ig constant regions of the light and heavy chains. For abbreviations, see the legend to Fig. 4

As for the constant region of the Ig light chains, it was mostly expressed in L6 CT CTX, although the percentage was much lower than that of Ig heavy chains (Fig. 5, Table S3). Regarding the variable regions, no transcripts of the *Ighj* genes were detected (Table S3), however, RNAs for various types of *Ighv* and *Igkv* were detected. The percentage of L2/3 IT ENTl cells (cortical layer 2/3 intratelencephalic-projecting glutamatergic neuron, entorhinal area, lateral part) expressing any of the light chain variable regions is 0.42% and the percentage of L5 PT CTX expressing any of the heavy chain variable regions is 0.19% (Fig. 5). Closer examination of the variable region revealed that *Ighv7-1* in the heavy chain variable region, and *Igkv1-35*, *Igkv2-105*, *Igkv6-20*, and *Igkv7-33* in the light chain variable regions are repeatedly detected (Fig. 6A and B) (Table S3). In addition, several cells express both variable and constant regions of the Ig heavy chain (Fig. 6C), and there were 20 cells expressing both heavy and light chain constant regions, 19 of which were L6 CT CTX cells (Fig. 6D). Although we could not find cells that express both light and heavy chain variable regions, we obtained a trend that the probability of expression of the light chain variable region is higher in cell types that express the heavy chain variable region. The expression rates of the light and heavy chain constant regions also tended to be high in these cell types (Fig. 5).

As for *Cd79a* (encoding Igα) and *Cd79b* (encoding Igβ), which cooperate with membrane-bound IgM, *Cd79a* was widely expressed, although the proportion of cells expressing *Cd79a* was less than 3% of each cell class (Fig. 4A, Table S4). In contrast, *Cd79b* was expressed only in the micro-PVM cluster. As for the receptors for secreted form IgM such as *Pigr*, *Fcmr*, and *Fcamr*, *Fcmr* was most frequently expressed in a variety of cells, although the proportion of positive cells in L6 CT CTX was about 2.5% (Fig. 4A).

### LPS stimulation causes reduction of *Ighm* expression in microglia

**Fig. 7:**
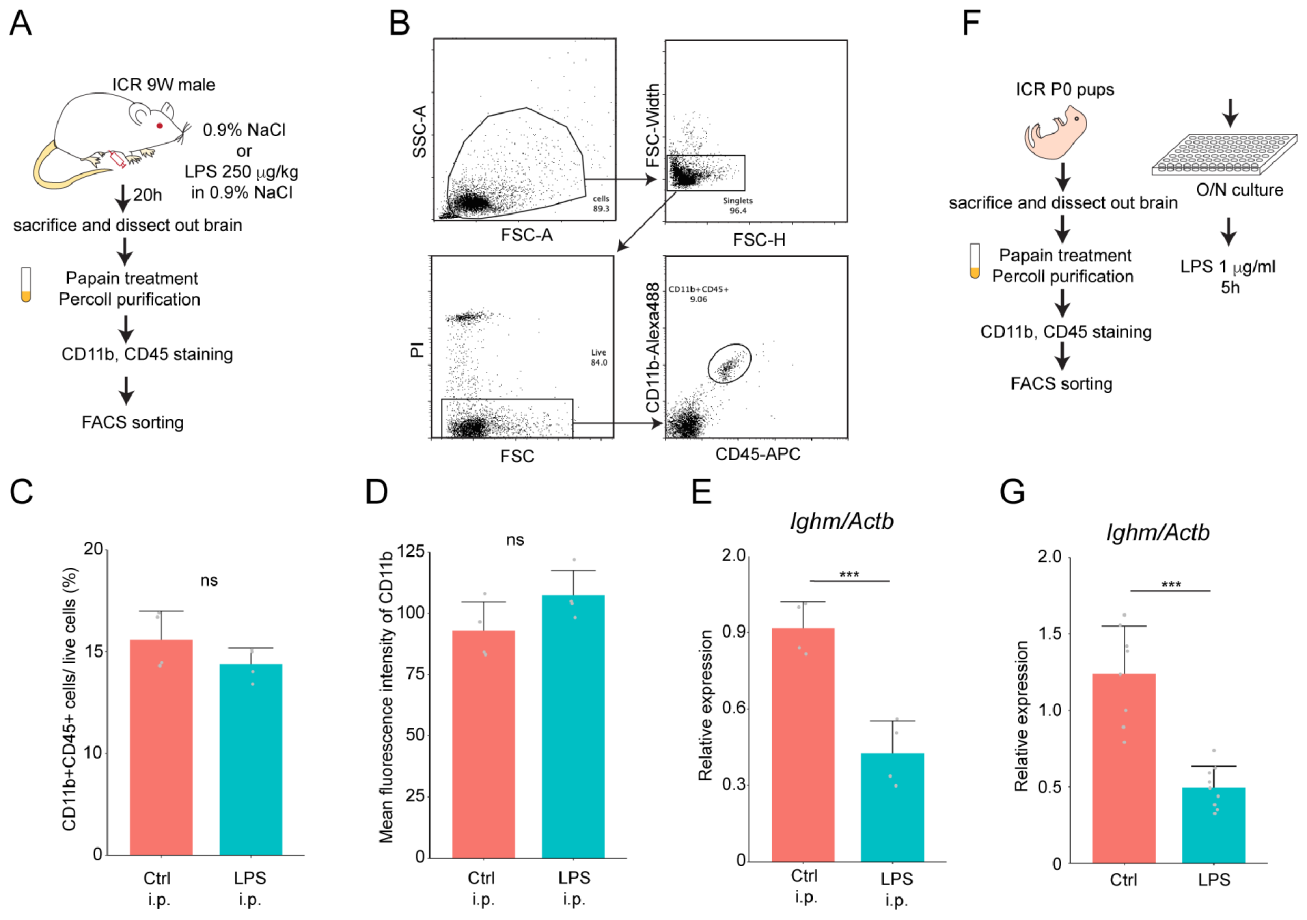
LPS effects on *Ighm* expression. **A**. Experimental scheme of microglia preparation after LPS challenge. **B**. FACS sorting strategy for CD11b + CD45 + microglia. **C**. Percentage of CD11b + CD45 + microglia among live cells was calculated. **D**. Mean fluorescence intensity of CD11b staining among microglia was examined. **E**. *Ighm* expression in microglia from Ctrl and LPS-injected mice was assessed by real time PCR. **C**.**D**.**E**. *n* = 4 for each condition. **F**. Experimental scheme of microglia preparation from P0 pups. **G**. *Ighm* expression in microglia after stimulation with 1 μg/ml LPS was measured by quantitative real-time PCR. Ctrl: *n* = 8, LPS: *n* = 9. ns: not significant, ***:<0.01

Since *Ighm* transcriptions increase after immune activation such as LPS stimulation in B cells [26–28], we next investigated the microglial response to LPS challenge. Adult mice were injected intraperitoneally with LPS and CD11b + CD45 + microglia were sorted by FACS after 20 h (Fig. 7A, B). The proportion of CD11b + CD45 + microglia was almost similar between saline-injected mice (Control, Ctrl) and LPS-injected mice (Fig. 7C). Microglia from LPS-injected mice tended to have increased surface expression of CD11b, although the difference was not statistically significant (Fig. 7D). However, the expression of *Ighm* was dramatically decreased in microglia from LPS-stimulated mice (Fig. 7E). To examine the direct effect of LPS stimulation on *Ighm* expression in microglia, CD11b + CD45 + microglia from P0 pups were sorted by FACS (Fig. 7F). After 5 h of LPS stimulation in vitro, *Ighm* expression in microglia was significantly reduced (Fig. 7G), indicating that LPS directly downregulates the *Ighm* expression in microglia.

## Discussion

This study investigated the expression of *Ig* genes in the developing and adult mouse brain. Using scRNA-seq data, we observed the expression of *Ighm*, which encodes the constant region of the IgM heavy chain, in microglia. This expression was pronounced during developmental stages and was confirmed by in situ hybridization. Further investigation revealed the presence of both membrane-bound and secreted forms of *Ighm* transcripts in microglia. Notably, the microglial *Ighm* transcripts lacked the V and D regions, which are important for ensuring Ig diversity. In addition to microglia, *Ighm* expression was also detected in specific neuronal populations, particularly in L6 CT CTX neurons in the adult brain, a fraction of which co-express both *Ighm* and *Ighg2c.* The detection rate of any of the heavy chain constant regions reached nearly 60% in L6 CT CTX, and the variable regions of the Ig light chain were detected in more than 0.4% of L2/3 IT ENTl. Finally, we found that *Ighm* expression in microglia is decreased after LPS stimulation.

There have been several reports on the expression of Igs in non-B cells [29]. For example, macrophages including tumor-associated macrophages, and circulating monocytes have been reported to express Igs with rearranged variable regions, although the diversity is quite limited [30–32]. To the best of our knowledge, the present study is the first report of the *Ighm* transcripts in microglia. Of note, more than 80% of embryonic-stage microglia and 35% of adult Micro-PVM expressed *Ighm*. It is difficult to compare the expression levels because they are from different databases, but the high expression rate of *Ighm* in microglia may suggest that they have some physiological significance. Since we were unable to detect V and D regions in microglia even by V(D)J repertoire analysis, it is likely that at least the majority of microglial *Ighm* transcripts lack the V and D regions, although the possibility that *Ighm* with variable regions exist cannot be completely ruled out. In the future, it is necessary to increase the number of samples and read the sequences more deeply, or search using multiple databases. As for the predicted translated products, the start codon is present in *Ighj4* for transcripts containing upstream of *Ighj4*, but there is no start codon until exon1 of *Ighm* for transcripts containing *Ighj1* or *Ighj3*. Therefore, when these transcripts are translated, they may contain part of the *Ighj4* region or a protein containing only the constant region of IgM. They would not be diverse and may have a different role than antibodies, where diversity is particularly important.

As for the neuron-derived *Ig* transcripts, it has been reported that *Ighm* is expressed in both excitatory and inhibitory neurons in the spinal cord as well as in the brain [9, 10, 23]. However, there is no information on changes in their expression during brain development or on the comprehensive identification of the subtypes of cells that express them. In addition, information is still lacking on whether they simultaneously express variable and constant regions of the heavy and light chains and whether they show diversity in their variable regions. By using the big data of adult scRNA-seq data, we detected *Ighm* transcripts in a variety of neurons, including both excitatory and inhibitory neurons, in addition to glial cells. The most dominant expression of *Ighm* was observed in L6 corticothalamic neurons of the cerebral cortex, reaching 56%. However, neural *Ighm* expression was barely observed in embryonic and P0 brains. In situ hybridization analysis revealed that neurons begin to express *Ighm* transcripts around 2 weeks postnatally (Fig. S3), suggesting that *Ighm* may be expressed when neurons mature.

In addition, proteins encoded by *Ighg1* and *Ighg2* have been detected in rat astrocytes [10, 12], and *Ighg3* RNAs have been reported in inhibitory neurons [8, 9]. In this study, approximately 1.5% of the cells in the adult database expressed more than two Ig heavy constant regions, with the combination of *Ighm* and *Ighg2c* being the most common (Fig. 4B and C). For the immune cells, the simultaneous expression of *Ighm* and *Ighg2c* could only occur when they undergo class switch recombination. Some neurons may be put into a state similar to class switch recombination in immune cells. Although variable regions of Ig heavy and light chains have been detected in the dorsal root ganglion neurons and astrocytes in the spinal cord [10–12], their diversity and frequency remain unclear. In the present study, 51 light chain variable regions and 32 heavy chain variable regions were detected mainly in neurons in the adult database. Interestingly, several variable regions were repeatedly observed: *Igkv4-69*, *Igkv4-70*, *Igkv6-14*, *Igkv6-17*, *Ighv1-62* and *Ighv1-80* were detected in E16.0-E18.0 database (Fig. 1E and F) as well as in the adult database (Fig. 6A and B), and *Igkv4-70*, *Igkv4-74*, and *Igkv6-13* were detected both in P0 cortical samples (Fig. 2B) and adult brain database (Fig. 6A and B). These results suggest that certain variable regions may be more commonly transcribed in the brain. Whether neuron-derived Ig transcripts in general include both variable and constant regions remains unclear, although we identified cells expressing both components of the Ig heavy chain (Fig. 6C). To address this, repertoire analysis should be extended to adult neuronal samples, as has been done with neonatal tissue. In contrast to microglia, neurons lacked detectable J regions in the scRNA-seq data, suggesting distinct Ig transcript profiles. In addition, detection sensitivity, the percentage of cells with at least one detected variable region from both heavy chain and light chains, has been reported to be 48–94%, even in B cells [33]. Considering the low expression level of Ig transcripts in the brain compared to B cells, careful consideration should be given to whether there is a complete form of Ig transcript in neurons with variable regions similar to those in B cells.

Co-expression of both Igα and Igβ, which are required for intracellular signaling of membrane-bound Ig, was not observed. This suggests that even if membrane-bound Ig proteins exist, they may use a different mode of signal transduction than that used in B cells. As for the receptors for the secreted form of IgM, *Fcmr* tends to be expressed in the *Ighm*-expressing cell clusters, suggesting that the neuron-derived *Ighm* product has some local function, although further studies are needed to determine if the neuron-derived Ig transcript is translated and functions as a protein.

LPS stimulation normally upregulates *Ighm* transcription and IgM protein secretion from B cells, but the response of microglia to LPS was the opposite. The decrease in *Ighm* expression after LPS stimulation has also been reported for macrophages [32], while the *Ighm* transcripts in microglia are different from those reported for macrophages. The finding that immune activation signals reduce *Ighm* expression in microglia may suggest that the *Ighm* plays a role in the physiological condition but not in pathological condition. Since transcriptional regulation has been suggested to be different between B cells and non-B cells, such as lung cancer and colon cancer cell lines and epithelial tumor stem cells that express rearranged Ig [34–36], it is also possible that a different mode of transcription is used in microglia. Further analysis is required to elucidate the functional implications *of Ighm* expression in microglia and other Ig functions in the brain.

## Electronic supplementary material

Below is the link to the electronic supplementary material.

Supplementary Material 1

Supplementary Material 2

Supplementary Material 3

Supplementary Material 4

Supplementary Material 5

Supplementary Material 6

## Data Availability

The raw sequence reads generated in this study are available in the DNA Data Bank of Japan (DDBJ) Sequence Read Archive (DRA) under accession number DRA011104. This submission includes experiments DRX245194-DRX245195 and runs DRR255476-DRR255477. The data are linked to BioProject accession PRJDB10794 and BioSample accession SAMD00258698. All code used in this study is available upon request.

## Abbreviations

ACA: Anterior cingulate area
Astro: Astrocytes
BBB: Blood-brain barrier
BCR: B cell receptor
Car3: Carbonic anhydrase 3
Chodl: Chondrolectin
CNS: Central nervous system
CR: Cajal-Retzius
CT: Corticothalamic-projecting glutamatergic cortical neuron
Ctrl: Control
CTX: Cerebral cortex
DAPI: 4,6-diamidino-2-phenylindole, dihydrochloride
DEG: Differentially expressed gene
DG: Dentate gyrus
D-PBS (-): Dulbecco’s Phosphate buffered saline
E: Embryonic day
Endo: Endothelial cell
ENT: Entorhinal area
ENTl: Entorhinal area, lateral part
ENTm: Entorhinal area, medial part
FACS: Fluorescence Activated Cell Sorting
FC: Fasciola cinereal
Ig: Immunoglobulin
IG: Indusium griseum
IHC: Immunohistochemistry
IMN: Immature neuron
IT: Intratelencephalic-projecting glutamatergic cortical neuron
L2: Cortical layer 2
L3: Cortical layer 3
L4: Cortical layer 4
L5: Cortical layer 5
L6: Cortical layer 6
Lamp5: Lysosomal-associated membrane protein family, member 5
LPS: Lipopolysaccharide
Meis2: Meis homeobox 2
Micro: Microglia
NN: Non-neuronal
NP: Near-projecting glutamatergic cortical neuron
OB: Olfactory bulb
Olig: Oligodendrocyte
P: Postnatal day
P2RY12: Purinergic receptor P2Y, G-protein coupled 12
PBS: Phosphate buffered saline
PBST: PBS containing 0.05% Triton X-100
Peri: Pericyte
PFA: Paraformaldehyde
PPP: Postsubiculum-presubiculum-parasubiculum
ProS: Prosubiculum
PT: Pyramidal tract
Pvalb: Parvalbumin
PVM: Perivascular macrophage
RHP: Retrohippocampal region
RSP: Retrosplenial area
RT: Room temperature
RT-PCR: Reverse transcription polymerase chain reaction
scRNA-seq: Single cell RNA-sequencing
SMC: Smooth muscle cell
Sncg: Synuclein Gamma
SSC: Saline-Sodium Citrate buffer
SSCT: Saline-Sodium Citrate buffer with 0.1% Tween 20
Sst: Somatostatin
STR: Striatum
SUB: Subiculum
TE: Telencephalic regions
TPE: Temporal association (Tea)-perirhinal (PERI)-ectorhinal (ECT)
UMAP: Uniform manifold approximation and projection
Vip: Vasoactive Intestinal Peptide
VLMC: Vascular leptomeningeal cell
