# Supplementary material for: Comprehensive analysis of immunoglobulin expression in the mouse brain from embryonic to adult stages
Source: J Neuroinflammation. 2025 Jun 9;22:153. doi: 10.1186/s12974-025-03457-9 (PMC12147291; doi:10.1186/s12974-025-03457-9)
Supplement: Supplementary file 5 — Supplementary Material 5 [file 12974_2025_3457_MOESM5_ESM.pdf]

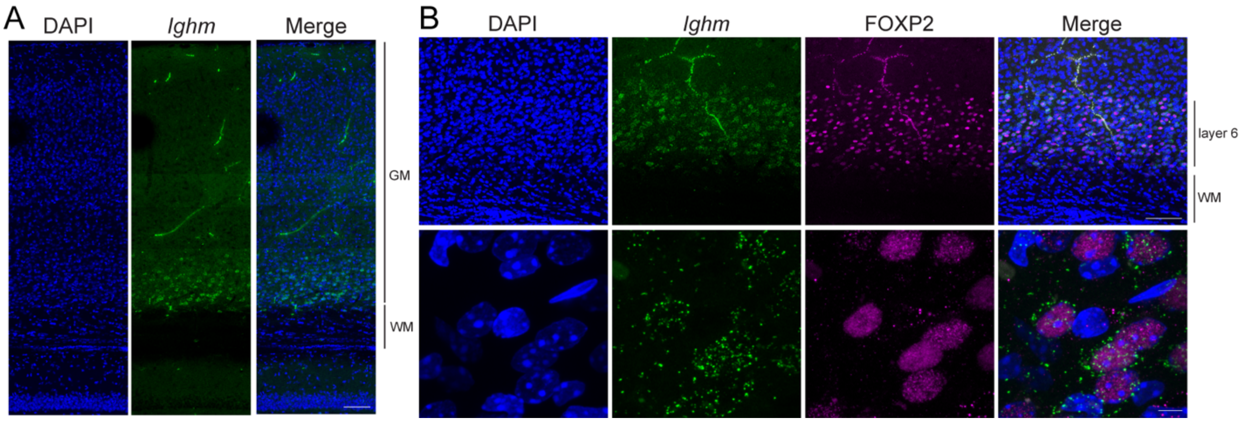

Supplementary Figure 1 | *In situ* hybridization for *Ighm* in the adult cerebral cortex.
A. *In situ* hybridization for *Ighm* on a section of cerebral cortex from 8W mouse. Scale bar = 75 µm. B. *In situ* hybridization for *Ighm* combined with IHC for FOXP2 on 8W mouse cerebral cortex. FOXP2-positive corticothalamic neurons in layer 6 express *Ighm*. Bottom panel is the magnification of the top panel. Scale bar = 75 µm (top panel), 5 µm (bottom panel). WM: white matter, GM: gray matter. n = 4 brains.

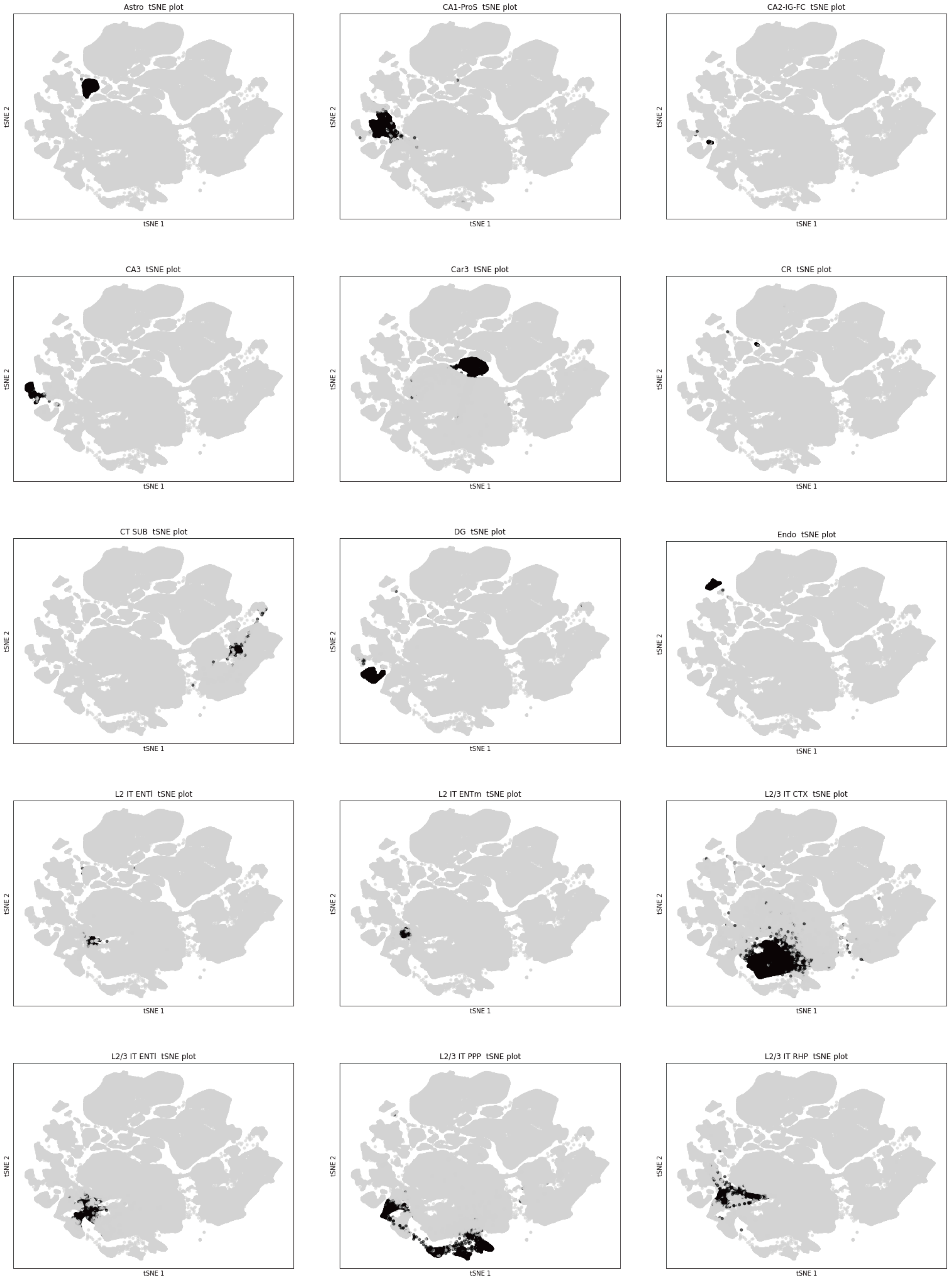
Astro tSNE plot
CA1-ProS tSNE plot
CA2-IG-FC tSNE plot
CA3 tSNE plot
Car3 tSNE plot
CR tSNE plot
CT SUB tSNE plot
DG tSNE plot
Endo tSNE plot
L2 IT ENTl tSNE plot
L2 IT ENTm tSNE plot
L2/3 IT CTX tSNE plot
L2/3 IT ENTl tSNE plot
L2/3 IT PPP tSNE plot
L2/3 IT RHP tSNE plot
tSNE 1
tSNE 2

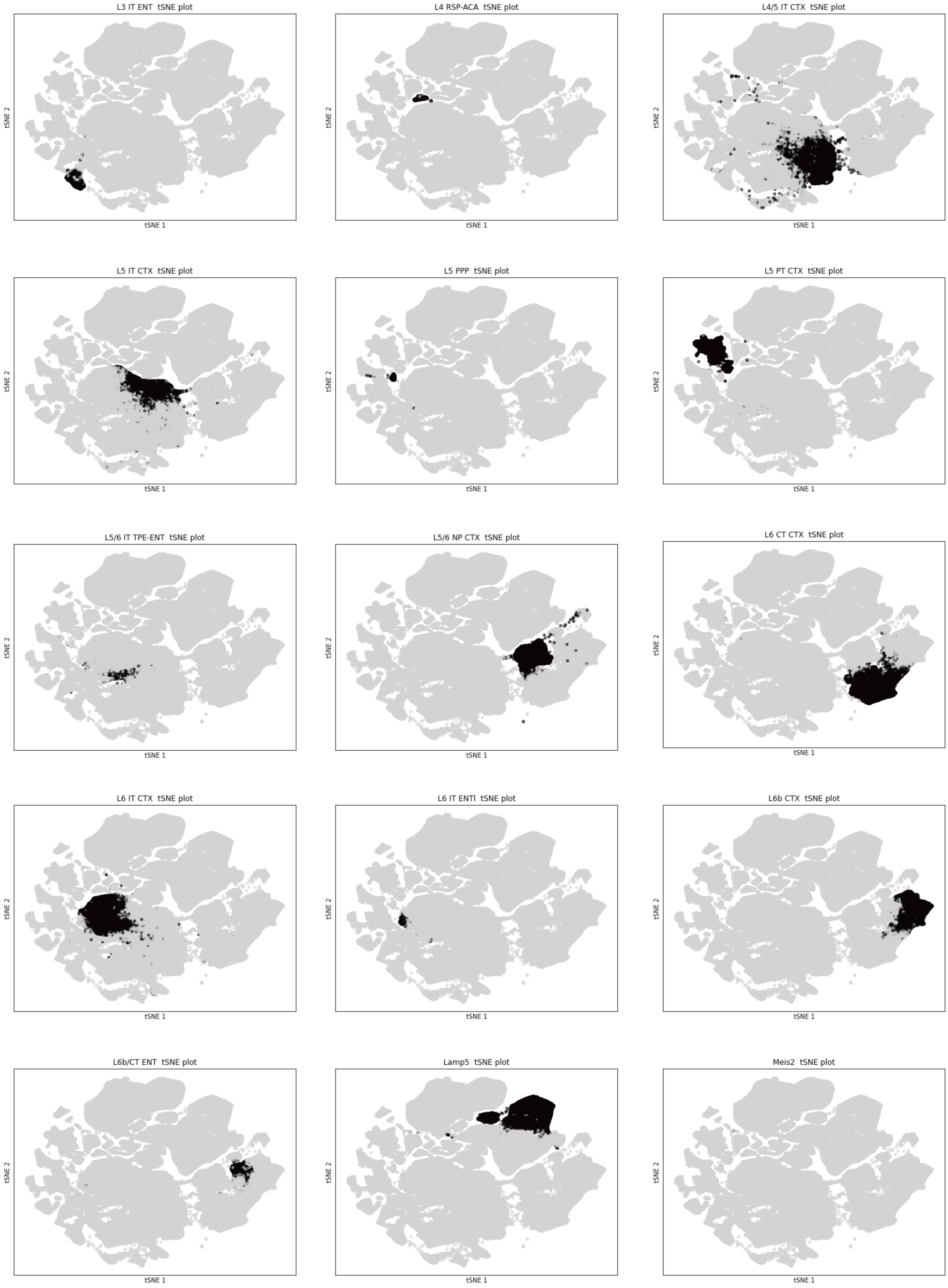
L3 IT ENT tSNE plot
tSNE 2
tSNE 1
L4 RSP-ACA tSNE plot
tSNE 2
tSNE 1
L4/5 IT CTX tSNE plot
tSNE 2
tSNE 1
L5 IT CTX tSNE plot
tSNE 2
tSNE 1
L5 PPP tSNE plot
tSNE 2
tSNE 1
L5 PT CTX tSNE plot
tSNE 2
tSNE 1
L5/6 IT TPE-ENT tSNE plot
tSNE 2
tSNE 1
L5/6 NP CTX tSNE plot
tSNE 2
tSNE 1
L6 CT CTX tSNE plot
tSNE 2
tSNE 1
L6 IT CTX tSNE plot
tSNE 2
tSNE 1
L6 IT ENTl tSNE plot
tSNE 2
tSNE 1
L6b CTX tSNE plot
tSNE 2
tSNE 1
L6b/CT ENT tSNE plot
tSNE 2
tSNE 1
Lamp5 tSNE plot
tSNE 2
tSNE 1
Meis2 tSNE plot
tSNE 2
tSNE 1

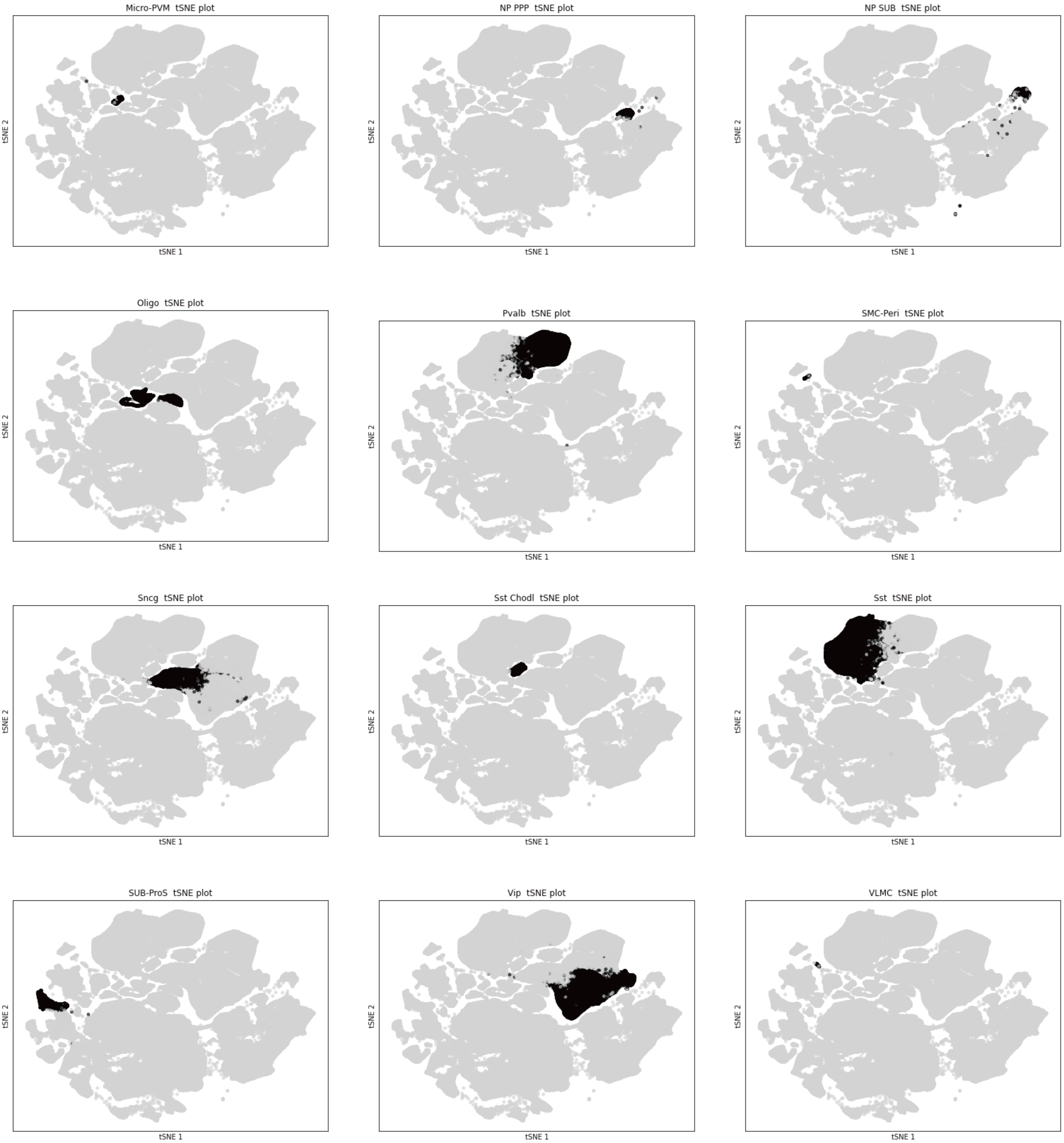
Micro-PVM tSNE plot
NP PPP tSNE plot
NP SUB tSNE plot
Oligo tSNE plot
Pvalb tSNE plot
SMC-Peri tSNE plot
Sncg tSNE plot
Sst Chodl tSNE plot
Sst tSNE plot
SUB-ProS tSNE plot
Vip tSNE plot
VLMC tSNE plot
tSNE 1
tSNE 2

Supplementary Figure 2 | Distribution of each subclass of cells illustrated on t-SNE plot. Each point represents an individual cell, and cells are color-coded according to their respective subclass. One specific subclass is highlighted in black for emphasis.

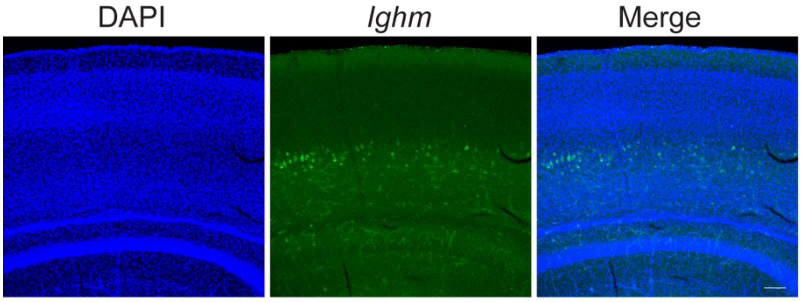

Supplementary Figure 3 | *In situ* hybridization for *Ighm* in P14 cerebral cortex.
A. *In situ* hybridization for *Ighm* on a section of cerebral cortex from P14 mouse. Scale bar = 100 µm. n = 2 brains.

Supplemental Table 1 | Details of Ig related genes expression in E16.0-E18.0 scRNA-seq data

Supplemental Table 2 | Details of Ig related genes expression in P0 scRNA-seq data

Supplemental Table 3 | Details of Ig related genes expression in adult scRNA-seq data

Supplemental Table 4 | Percentage of Ig related genes expression related to Figure 4
