# Supplementary figures and images for: Comprehensive analysis of immunoglobulin expression in the mouse brain from embryonic to adult stages
Source: J Neuroinflammation. 2025 Jun 9;22:153. doi: 10.1186/s12974-025-03457-9 (PMC12147291; doi:10.1186/s12974-025-03457-9)

Uncropped gels of Fig. 3B

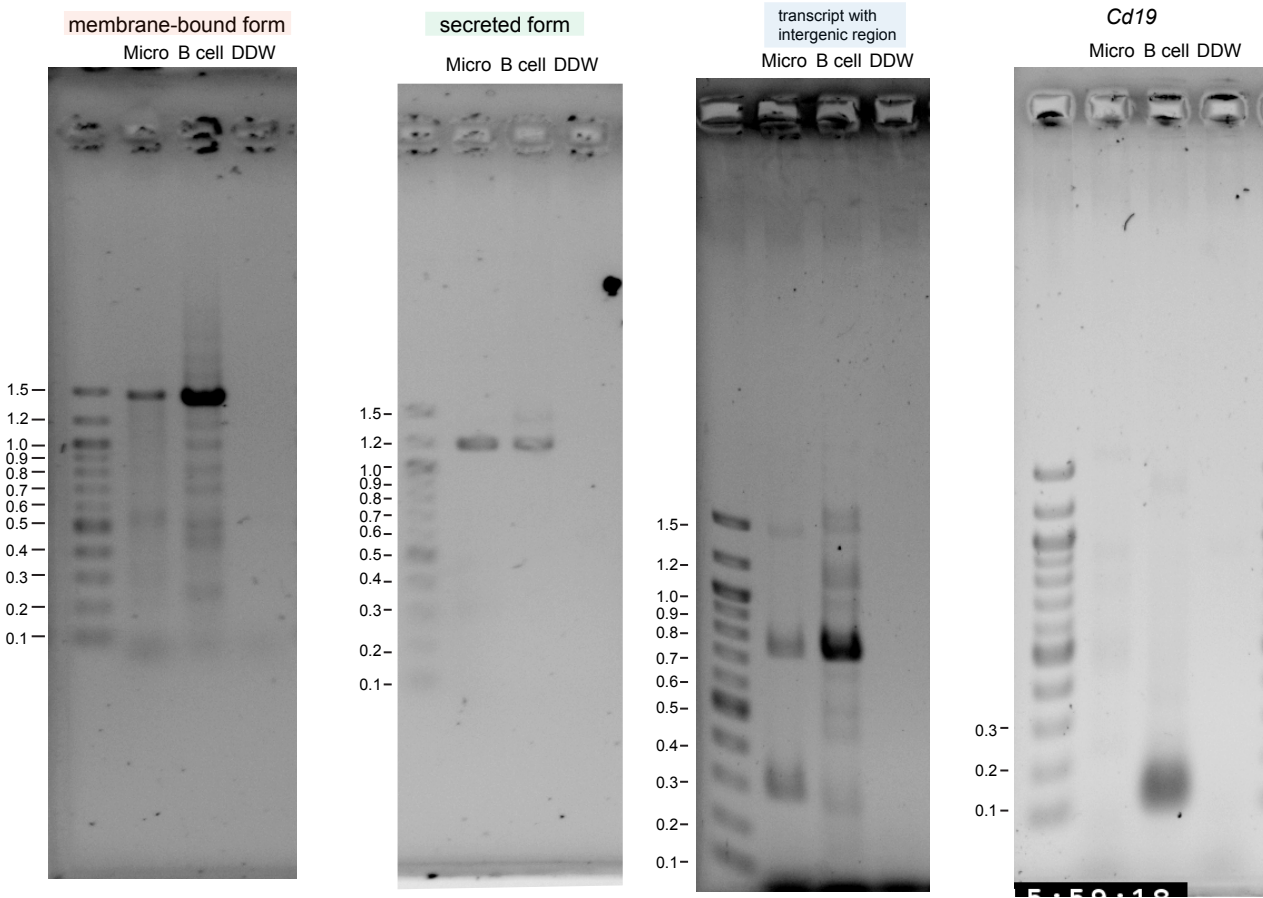

Supplement: Supplementary file 6 — Supplementary Material 6 [file 12974_2025_3457_MOESM6_ESM.pdf]
